# Relationship between recombinant protein expression and host metabolome as determined by two-dimensional NMR spectroscopy

**DOI:** 10.1371/journal.pone.0177233

**Published:** 2017-05-09

**Authors:** Young Kee Chae, Seol Hyun Kim, John L. Markley

**Affiliations:** 1 Department of Chemistry, Sejong University, Seoul, Korea; 2 Department of Biochemistry, University of Wisconsin – Madison, Wisconsin, United States of America; Scuola Internazionale Superiore di Studi Avanzati, ITALY

## Abstract

*Escherichia coli* has been the most widely used host to produce large amounts of heterologous proteins. However, given an input plasmid DNA, *E*. *coli* may produce soluble protein, produce only inclusion bodies, or yield little or no protein at all. Many efforts have been made to surmount these problems, but most of them have involved time-consuming and labor-intensive trial-and-error. We hypothesized that different metabolomic fingerprints might be associated with different protein production outcomes. If so, then it might be possible to change the expression pattern by manipulating the metabolite environment. As a first step in testing this hypothesis, we probed a subset of the intracellular metabolites by partially labeling it with ^13^C-glucose. We tested 71 genes and identified 17 metabolites by employing the two-dimensional NMR spectroscopy. The statistical analysis showed that there existed the metabolite compositions favoring protein production. We hope that this work would help devise a systematic and predictive approach to the recombinant protein production.

## Introduction

*Escherichia coli* is the organism of choice when it comes to the recombinant protein production [1]. Despite its weakness as a prokaryote in producing eukaryotic proteins, *E*. *coli* has many advantages: We can grow *E*. *coli* cultures easily within a short time at a low cost [2]. Its genetics is well known, and various strains have been developed for specific purposes. The usual protocol for producing proteins in *E*. *coli* cells includes the cloning of a target gene in an expression plasmid, transformation of a suitable host strain, and then the induction of the target protein synthesis. In theory, this is fairly straightforward, but in practice, there are many things to consider. When things go wrong and no protein is produced or only inclusion bodies are formed, one needs to consider many factors that could influence the transcription and translation of the target gene, including the promoter, terminators, operators, copy numbers for the DNA/plasmid construct, Shine-Dalgarno sequence, rare codons, mRNA secondary structure, and mRNA stability [3]. Furthermore, even if a large amount of the target protein is produced in its soluble form, the activity of the purified protein is not guaranteed. To deal with proteins that were difficult to express, researchers have developed various methods: strains able to translate rare codons [4], strains able to form disulfide bonds in the cytosol [5], plasmids with a special promoter [6], various fusion partners or tags [7–12], expression at a low temperature [13], secretion to periplasm [14], co-expression of chaperones [15–18], and genome engineering [19]. One or more of these measures may work, but finding the right one is usually a laborious and time-consuming process because it is all based on trial-and-error.

The intracellular composition of metabolites reflects the cell’s physiology at a particular time point. When the production of the target protein is induced, *E*. *coli*’s cellular physiology is perturbed in such a way that the metabolites are redirected from the normal pathway to meet the newly initiated need, and this process of adjusting the metabolic flux is similar to the response to the environmental stress [20]. In addition to the general response, there are specific responses to each given stress, indicating that a unique set of intracellular metabolites is needed to cope with it. If we perturb the metabolic environment in a cell by applying a stress, it may alter the expression pattern of the target protein.

In monitoring the metabolite profiles, NMR spectroscopy has been proven useful because of its ability to identify and quantify all major components in a high-throughput fashion [21]. For this kind of mixture analysis, one-dimensional (1D) ^1^H NMR has been extensively used alone or in combination with chromatographic techniques such as LC-NMR and GC-NMR. One-dimensional NMR is fast, but suffers from inevitable signal overlap, and recently, two-dimensional (2D) ^1^H-^13^C NMR has become an alternative in terms of higher accuracy and comparable data collection time [22]. The isotope labeling of metabolites with ^13^C vastly enhances the sensitivity of 2D ^1^H-^13^C NMR and reduces the data collection time.

Our hypothesis is that the intracellular metabolic state *E*. *coli* cells engaged in successful protein expression will differ from those of *E*. *coli* cells for which the process is unsuccessful. If this is the case, then it might be possible to manipulate the expression pattern by changing the intracellular metabolite profile through a combination of exogenous stresses. We regard *E*. *coli* as a kind of machine that accepts a plasmid as an input and outputs a target protein. We observe and record its metabolic state after it is given an induction signal. If it malfunctions, then we apply a proper stress and adjust the environment so that it functions. To do so, we first need to define a region in the metabolite space where the protein production is not favorable. We also need to estimate the effects of various stresses on the metabolite compositions so that we can predict the direction along which a metabolite state would move due to a specific stress. How far it will move will depend on the strength of the stress. Then, for a case of no expression, we can predict which stresses and how much of them are needed to move it out of the unfavorable region. In this way, we can bypass the trial-and-error process because we already know the proper stresses to apply to provide a favorable metabolite environment for a successful protein production. In addition to applying stresses, we anticipate that we will also supply necessary metabolites to optimize the condition if we can sort out key metabolites that are prominently present in the cases of high expression. The metabolite composition would be the result of the protein production, but it would also be thought as an environment for protein production.

What happens to the intracellular metabolites after the induction signal is given? Are there any metabolite differences between successful and poor productions? If so, how are we going to use this information? These are the questions that we’d like to answer at this initial stage of the systematic optimization of the recombinant protein production.

## Materials and methods

### *E*. *coli* growth

The expression plasmids were obtained from the Center for Eukaryotic Structural Genomics (Madison, WI, USA). We used 71 genes from 4 workgroups chosen at random: WG2137, WG2399, WG3743, and WG4122. Genes in WG2137 were cloned into expression plasmid pVP16 [23], and the genes of the other three workgroups were cloned into pVP68K [24]. All genes were fused to DNA coding for the cleavable expression tag MBP (maltose binding protein), and a TEV or 3C protease cleavage site was inserted between MBP and the target protein. Of the 71 genes, 19 were from *H*. *sapiens*, 4 from *C*. *elegans*, 6 from *M*. *musculus*, 16 from *S*. *cerevisiae*, 4 from *D*. *rerio*, 7 from *C*. *merolae*, 11 from *A*. *thalinana*, and 1 each from *X*. *laevis*, *R*. *norvegicus*, *P*. *pyralis*, and *G*. *sulphuraria*. Details of the genes used in this study can be found in the S1 File.

Rosetta2(DE3)/pLysS (Novagen, Madison, WI, USA) cells harboring the expression plasmid was grown overnight at 37°C in 2 mL LB medium (1% bactotrypton, 0.5% yeast extract, 1% NaCl) supplemented with 200 μg/mL ampicillin and 34 μg/mL chloramphenicol for WG2137 or 50 μg/mL kanamycin and 34 μg/mL chloramphenicol for WG2399, WG3743, and WG4122. The next day, 2 mL of fresh OvernightExpress Instant LB medium (Merck Millipore, Darmstadt, Germany) with the same antibiotics and 1% (w/v) of ^13^C glucose was inoculated with 1 μL of the overnight culture, and the culture was allowed to grow further for another 18 h. At harvest, the optical density at 600 nm (OD_600_) was measured with a Scinco S-3100 spectrophotometer (Scinco, Seoul, Korea). An aliquot of 0.1 mL of the culture was harvested and reserved for SDS-PAGE analysis. The other aliquot of 1 mL of the culture was harvested for preparation of the NMR sample. The cells for NMR samples were washed 3 times with 1 mL PBS buffer (10 mM sodium phosphate buffer at pH 7.4 with 150 mM NaCl) and frozen at -80°C.

### Protein expression analysis

To each cell pellet from 0.1 mL culture as mentioned above, 100 μl of BugBuster MasterMix (Merck Millipore, Darmstadt, Germany) was added. After incubation at 37°C for 10 min, 50 μl were taken and mixed with the same volume of 2x SDS-PAGE sample buffer. The expression profile was analyzed by SDS-PAGE (ExpressPlus PAGE gel 4–20%; Genscript, Piscataway, NJ, USA).

### Metabolite extraction and NMR sample preparation

Metabolites were extracted by the modified boiling water method [25]. First, the frozen cells in the 1.7 mL microtubes were lyophilized. The lyophilized cell pellet was ground into fine powder, and 0.6 mL of NMR buffer (5 mM HEPES, 0.2 mM DSS, 0.5 mM NaN_3_ in D_2_O) was added. The mixture was incubated at 100°C for 10 minutes in a hot block. Samples were cooled down on ice, and then centrifuged at 14000 RPM at room temperature for 10 minutes. The supernatant was transferred to a new microtube, and the pH was adjusted to 7.38 ± 0.02 using deuterium chloride or sodium deuteroxide.

### NMR data collection and data processing

All experiments were performed at 298 K on Bruker Avance II 500 MHz with a TXI probe (Bruker BioSpin GmbH, Rheinstetten, Germany) installed at Sejong University. Sensitivity enhanced ^1^H-^13^C HSQC spectra were collected with 16 scans per transient. The raw data contained 1024 and 40 complex points in the *t*_2_ and *t*_1_ dimensions, respectively. The spectral widths were 10 ppm for ^1^H and 115 ppm for ^13^C. The carrier frequencies for ^1^H and ^13^C were set at 4.7 ppm and 55 ppm, respectively. The DSS (4,4-dimethyl-4-silapentane-1-sulfonic acid) resonance was used as the chemical shift reference. The raw data were processed with NMRPipe [26]. For the ^1^H dimension, a squared cosine window function and zero-filling were applied sequentially, followed by a proper phase correction. For the ^13^C dimension, a linear prediction, a squared cosine window function and zero-filling were applied sequentially, followed by the zeroth order phase correction of 90 degrees. The final spectrum contained 2048 and 256 real points in *f*_2_ and *f*_1_, respectively. The resulting spectra were visualized and analyzed by rNMR [27]. The peaklist generated by rNMR was sent to MMCD (http://mmcd.nmrfam.wisc.edu) [28], PRIMe (http://prime.psc.riken.jp) [29, 30], and ECMDB (http://www.ecmdb.ca) [31, 32] to retrieve a list of candidate metabolites that might exist in the sample. Only the metabolites that could be confirmed with the actual HSQC spectra of standard compounds were retained to create the resonance intensity table. The resonances with no overlap with others were selected as representatives of the corresponding metabolites. The intensity table with those representative resonances was created by rNMR. In-house developed software [33, 34] was used first to normalize the intensity data to one of the resonances of HEPES (^1^H = 2.94 ppm, ^13^C = 54.69 ppm), the internal standard, then to compensate for the optical density differences between samples, and finally to yield a proper output format for the multivariate analysis. Multivariate analysis was performed with the R statistics software package (http://www.r-project.org) or MetaboAnalyst 3.0 [35]. The script for generating PCA scores plots and biplots by R was first written and provided by Ian Lewis (University of Calgary, Calgary, Canada) and further modified in-house [36, 37].

## Results and discussion

### Sample preparation

Although LB broth is a rich medium, it is carbohydrate deficient, and *E*. *coli* must utilize amino acids or peptides as a carbon source [38]. The LB broth being carbohydrate deficient was well suited to our strategy to label metabolites with the input ^13^C glucose. We examined the degree of labeling with 0.5, 1.0, and 2.0% ^13^C glucose, and observed that 1.0% glucose yielded a spectrum with signal intensity nearly equal to that from 2.0% glucose (data not shown). We were also careful not to disturb the optimized composition of OvernightExpress medium for protein production, which was the major reason for choosing 1.0% glucose rather than 2.0% glucose.

### Protein expression analysis

At first, we used a normal LB medium with IPTG induction at OD_600_ = 1.0. However, with many samples to manage, it became difficult to check whether OD_600_ of each sample reached 1.0. Since we were to compare the metabolite profiles of different target proteins, it was desirable to make the condition of every sample as uniform as possible, and in that sense, IPTG induction was not a good fit. Studier’s autoinduction medium [39] was another option to choose, but we thought that it would take time before it was established soundly in our lab. The OvernightExpress LB medium was the method of choice for our given situation.

The protein expression pattern was monitored by SDS-PAGE. Many samples showed clear cut patterns so that we could label without difficulty: “S” for a soluble protein, “I” for an inclusion body, and “N” for no expression. However, others showed mixed patterns: some samples showed comparable bands at both “I” and “S”, and some showed weak but discernable bands at “S”. We labeled such cases “S” if we could observe reasonable amounts of soluble protein. Of the 71 genes tested, 26 yielded soluble protein, 24 inclusion bodies, and 21 no expression. As for the plasmid dependence of the expression pattern, we found 2 soluble proteins and 8 inclusion bodies, and 16 no expressions for pVP16 (WG2137, ampicillin resistance), which contrasted with 24 soluble proteins, 16 inclusion bodies, and 5 no expressions for pVP68K (WG2399, WG3173, and WG4122, kanamycin resistance). Since we did not shuttle the genes from pVP16 to pVP68K, or vice versa, it is hard to say that pVP68K or kanamycin resistance was superior to pVP16 or ampicillin resistance in producing soluble protein. Rather, we think that many of the difficult genes happened to be in WG2137 under ampicillin. We also checked the molecular size dependence of the expression pattern. The genes were all fused to the maltose-binding protein (MBP), whose size was approximately 42.5 kDa. The size of the target proteins ranged from 9.7 to 72.5 kDa. For 38 proteins smaller than 30 kDa, there were 7, 13, and 18 cases of inclusion body, no expression, and soluble expression, respectively. For 33 proteins larger than 30 kDa, there were 17, 8, and 8 such cases. It seems that the ratio of soluble expression decreases as the molecular size increases, which may imply a limit to the solubilization ability of MBP. However, there were fewer samples with no expression as molecular the size increased.

It appears that the pattern of the protein production depends on the genes themselves, not on the nature of the plasmids. Changing plasmids sometimes leads to successful production of soluble proteins, but there are many other things to consider such as copy number, selection marker, promoter, terminator, operator, tag, fusion partner, etc [1]. The current approach to protein production relies on trial-and-error, and in many cases, luck is a non-trivial factor.

### NMR data collection and processing

Experimental errors could have come from any stage between the metabolite extraction and NMR instrumentation. Very minor things, such as pipetting and transferring an NMR sample from a microtube to an NMR tube, can be a source of a random error because the volume difference can change the parameters of the NMR instrument. We think that a major part of the experimental error comes from the pH adjustment of the NMR sample: when the pH was adjusted, different amounts of DCl and/or NaOD were added for different samples, which could result in differences first in volume and second in salt concentration. Adding different amounts of DCl or NaOD is due not only to the skill of a researcher but also to the concentration of each sample. The signal intensity is directly proportional to the metabolite concentration, but is lowered by the increasing salt concentration, both of which contribute to the NMR experimental error. We kept track of the amount of added DCl or NaOD to compensate the volume increase. The errors coming from NMR instrumentation due to many factors including the difference in salt concentration could be suppressed by using the internal standard, HEPES, which was contained in all NMR samples at the same concentration, 5 mM. One of the HEPES signals was selected as a reference [40], and the intensities of the metabolite signals in each spectrum were multiplied by a factor that could make the intensity of the reference signal in every sample the same [41]. The cell density differences were compensated by dividing intensities by OD_600_ which was assumed to be proportional to the number of cells. The final OD_600_ of samples were mostly around 15, but some were over 20. However, the final cell density did not look correlated with the expression pattern.

### NMR data analysis

**Fig 1:**
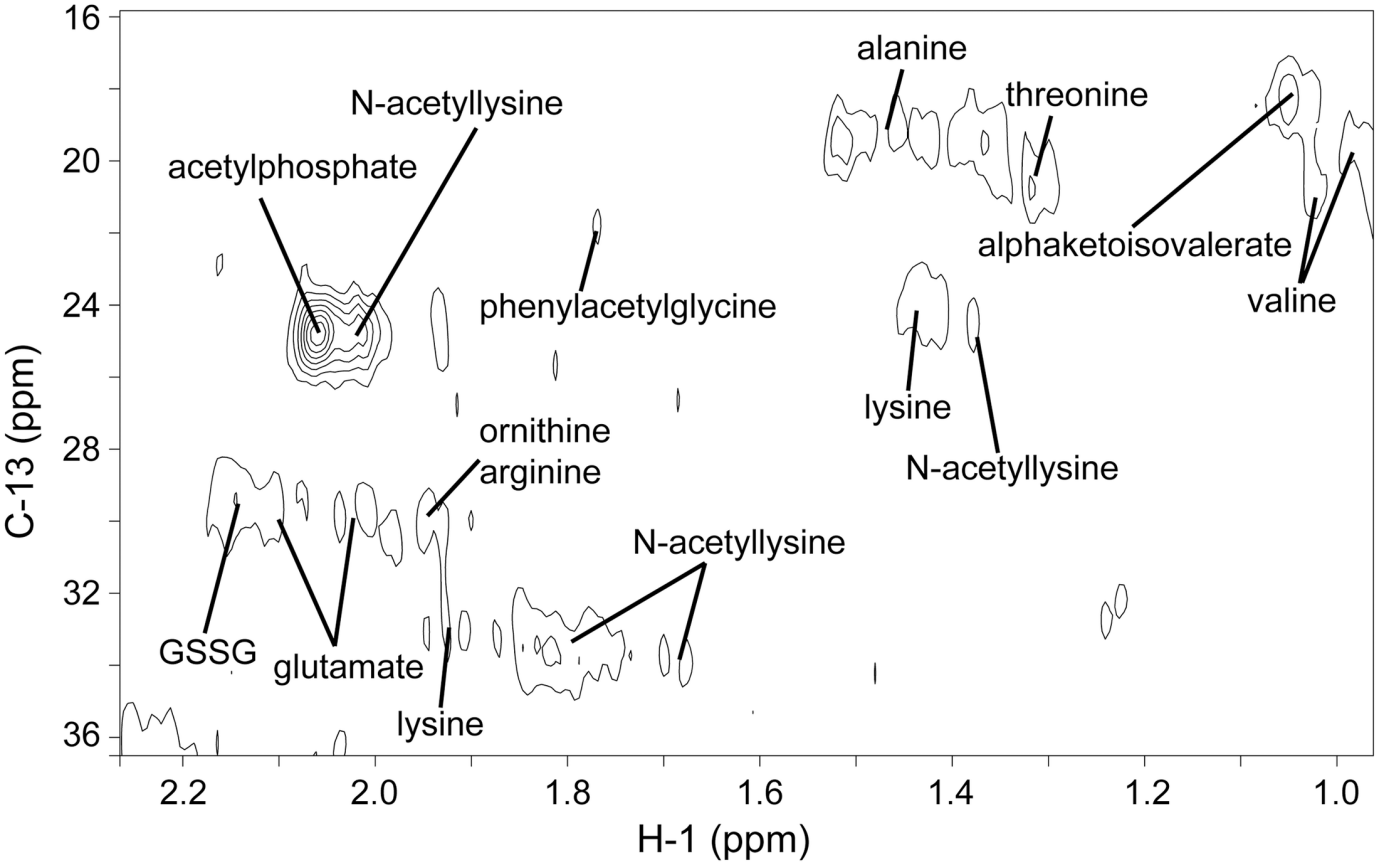
Low-frequency region of the two-dimensional ^1^H-^13^C HSQC spectrum of a sample (A1 of WG2137). Assigned resonances are labeled.

Fig 1 shows the low-frequency region of the NMR spectrum of a sample (A1 of WG2137) along with the assignments of the identified metabolites. By consulting three public databases and confirming with the standard spectrum library, 17 metabolites were identified, including 10 amino acids/derivatives (alanine, arginine, glutamate, lysine, threonine, valine, N-acetyllysine, ornithine, oxidized glutathione, and phenylacetylglycine), 4 organic acids (α-ketoisovalerate, acetylphosphate, succinate, and betaine), 1 alcohol (glycerol), 1 sugar derivative (amygdalin), and 1 nucleic acid (NAD). Glycerol and betaine are well known osmoprotectants. Glycerol has been reported to act as a housekeeping metabolite in the sense that it is present at high concentrations [42, 43]. Oxidized glutathione was observed, but the reduced form was not detected, which would imply that the protein production and/or the stationary phase depleted the reduced form since the glutathione is known to be almost completely reduced in the normal *E*. *coli* cells [44]. α-ketoisovalerate is a branched chain organic acid which is a precursor as well as a degradation product in leucine and valine metabolism, and it enters the central carbohydrate metabolic pathway through pyruvate. Acetylphosphate can be generated from pyruvate by pyruvate oxidase and converted to acetyl-CoA. Glutamate is a central amino acid that can be converted to and from many other amino acids, and can also be metabolized to succinate through γ-aminobutyric acid. We did not observe any other metabolites of the TCA cycle, which might imply the source of succinate was glutamate which had been converted from the input ^13^C-glucose. Citrate and malate were the other two intermediates of TCA cycle that have been observed in many of cases [25, 33, 34, 36, 37, 41–43], but not in this work.

### Multivariate analysis

**Fig 2:**
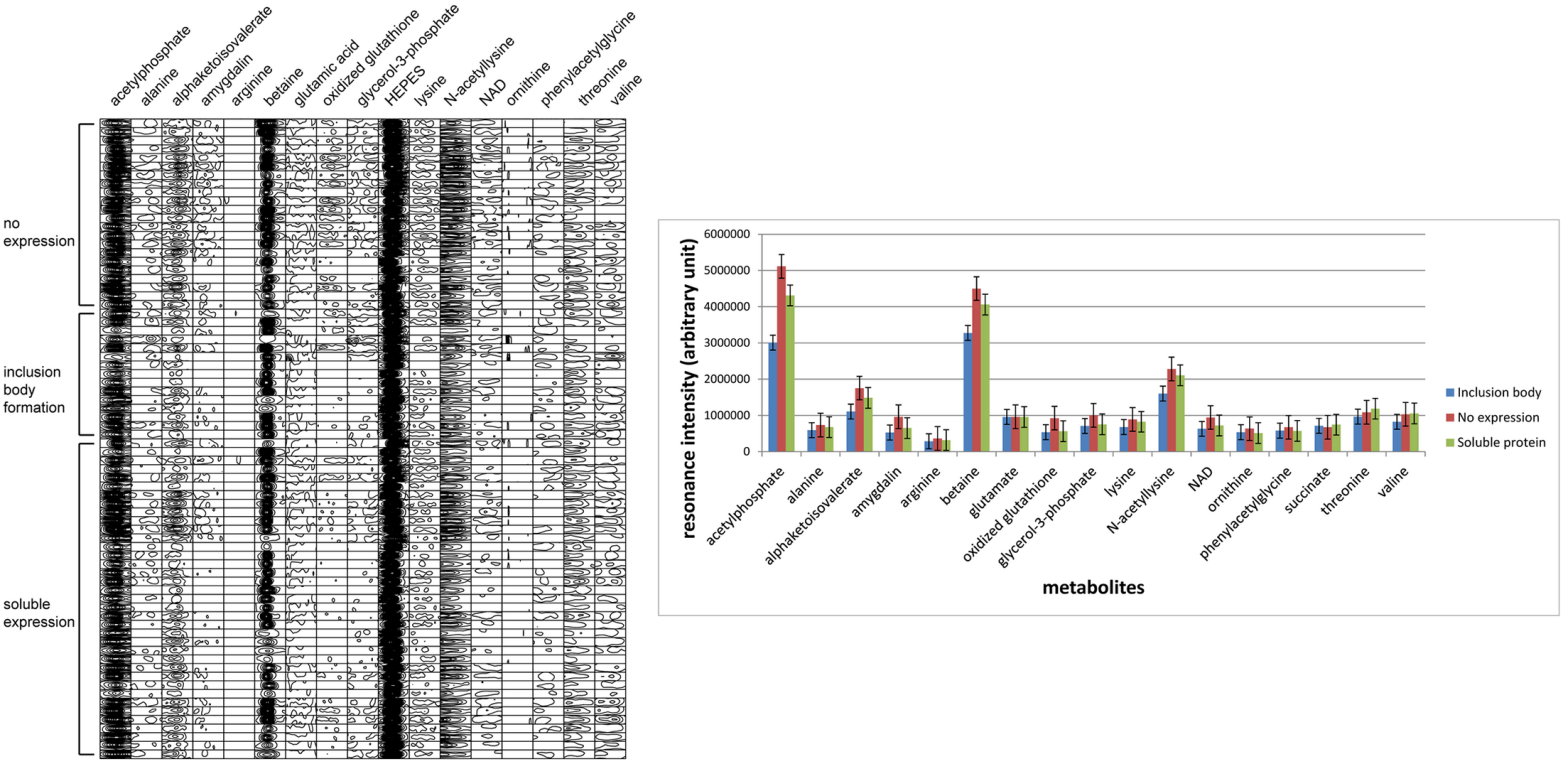
**(a) ROI view of the representative resonances of identified metabolites. (b) Barchart representation of ROI view.** Averages of metabolites within each group were converted to vertical bars with corresponding standard error bars.

Fig 2a shows the regions-of-interest (ROIs) of the spectra of all samples. The resonances were chosen in such a way that they were not overlapped with any others. Fig 2b shows a barchart representation of Fig 2a. Acetylphosphate, betaine, N-acetyllysine, and α-ketoisovalerate showed a pattern: they were the highest in the cells with no expression, and the lowest in those with inclusion bodies, while cells with soluble proteins were in the middle, closer to those with no expression. N-acetyllysine and α-ketoisovalerate are the intermediates in the degradation pathway of lysine and valine. It seems that their consumption was beneficial to target protein production, but too much consumption led to inclusion body. The tuning of their consumption to a certain level may be the key to the soluble protein production. This interpretation is also true for acetylphosphate and betaine. Betaine is a well-known osmoprotectant, but too much of it would favor the inclusion body formation, which seems reasonable because the concentration of total solutes inside the cell would decrease as the inclusion body forms and betaine would help cells to cope with such situation. Acetylphosphate to acetyl-CoA conversion would help meet the energy demand for protein production, so its accumulation implies that the protein is not produced efficiently while its fast consumption is linked to inclusion body formation. It can react with lysine to form N-acetyllysine, which is less plausible because both compounds showed elevated levels. Before the cells reach the stationary phase, that is, the induction signal, the composition of intracellular metabolites would be very similar in all three cases. *E*. *coli* cells in LB broth have been reported to depart from the steady state when OD600 ~ 0.3 [38]. After they enter the stationary phase, the non-expressing cells would experience metabolite changes due to the stationary phase itself, not to the stress of expressing proteins: both would be experienced by the cells of the soluble expression and inclusion body formation. It looks that forming an inclusion body drives the overall reaction to the direction of protein production by precipitating it continuously out of the solution state of the intracellular fluid until the harvest or the depletion of the resource, making it more distant from the non-expressing case. In case of soluble expression, the metabolite composition would be in between the other two. Since we did not measure the absolute concentrations of those metabolites, it was not possible to find out which contributed more to the expression pattern, the relative composition of each metabolite or the large absolute concentration changes of key metabolites. We performed multivariate analysis based on the relative changes of all metabolites, in a hope to view the holistic profile of the cells of those 3 expression patterns.

**Fig 3:**
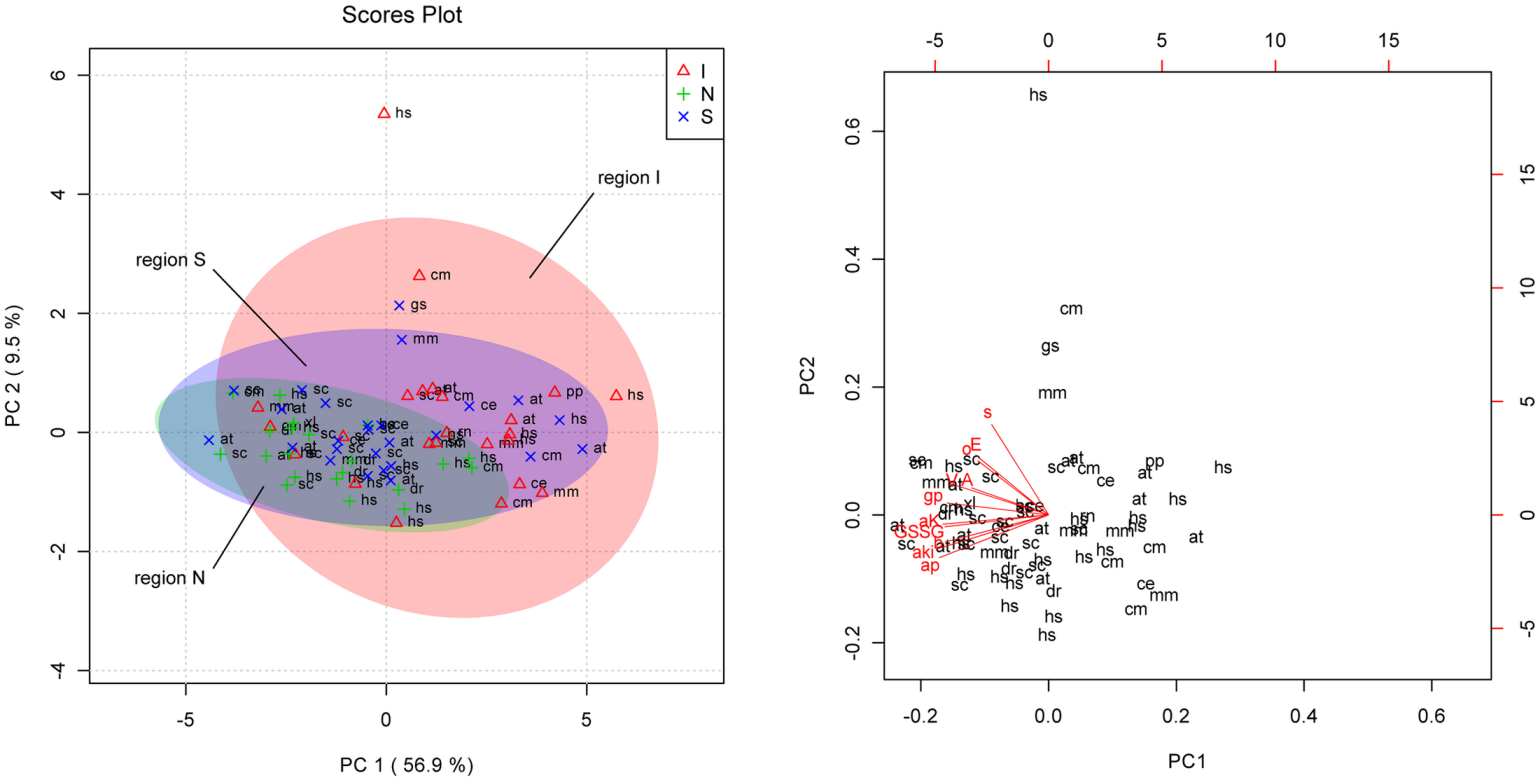
**(a) PCA scores plot.** I (triangle), N (+), and S (x) represent samples expressing inclusion bodies, none, and soluble proteins, respectively. The source of the sample was denoted with 2-letter abbreviations: at, *Arabidopsis thaliana*; ce, *Caenorhabditis elegans*; cm, *Cyanidioschyzon merolae*; dr, *Danio rerio*; gs, *Galdieria sulphuraria*; hs, *Homo sapiens*; mm, *Mus musculus*; pp, *Photinus pyralis*; rn, *Rattus norvegicus*; sc, *Saccharomyces cerevisiae*. **(b) Biplot along the first and second principal component axes.** For better visibility, only the following metabolites were selected to avoid crowdedness: A, alanine; aK, N-acetyllysine; aki, alphaketoisovalerate; ap, acetylphosphate; b, betaine; GSSG, oxidized glutathione; gp, glycerol-3-phosphate; o, ornithine; V, valine.

Fig 3a and 3b show the PCA scores plot and the biplot, respectively. For convenience, the regions encompassing samples of no expression, inclusion bodies, and soluble proteins were designated as “N”, “I”, and “S”, respectively. The “N” region contains all 3 patterns, and the “S” region, soluble proteins and inclusion bodies. We could observe several interesting features in Fig 3a. First, “N” region was the most compact, implying that the metabolic situations in the cells with no expression were quite uniform. This is reasonable because they all experienced the stationary phase, but presumably not the stress coming from the protein production. That is, the “N” region was composed of the samples without proteins expressed, making it the least dependent on the identities of genes. However, this is not entirely true because the soluble proteins and inclusion bodies were also present in the “N” region, which indicated that such proteins did not perturb much the metabolite pool of the host when they were produced even in a large amount. Second, all 3 regions overlapped which contradicted our hope to observe 3 separate clusters of each region. Out of 71 samples, only 21 were located outside the “N” region, implying that the majority of the metabolic situations in the 3 cases were similar. Those 21 samples include 7 of 26 expressed soluble proteins, and 14 of 24 inclusion bodies. This correlates well with the barchart since “S” samples were closer to “N” than “I”. According to the biplot in Fig 3b, the levels of all metabolites are higher (or more accumulated) in the “N” region. The metabolites being more accumulated does not mean that the target protein is not expressed because we have proteins expressed in their soluble or insoluble forms in the “N” region, too. Rather, it designates a region where a possibility of protein expression is lower. To get a higher probability of protein expression, the metabolite profile should be moved out of the “N” region, and this could be done by applying exogenous stresses. It is our hypothesis that *E*. *coli* can produce the target protein in its desired form if it is provided a suitable metabolic environment.

The ultimate goal of this work was to establish a systematic way to produce a target protein in a desirable form, eliminating the need to try various methods through a trial-and-error fashion. Our hypothesis started from the assumption that the metabolite compositions resulting from desirable productions of target proteins would be similar to one another while those from unsuccessful ones would also be similar among themselves. Our main hypothesis is that the metabolite composition provides an environment for protein production (as well as the former is the result of the latter), and if we change the metabolite composition, then we can change the expression pattern. The metabolite composition, in turn, can be changed by applying external stresses. We found a relationship between expression pattern and the metabolite composition regardless of the construct of target proteins (fusion or tag) or their overall sequences. We regard *E*. *coli* as a black box which takes a plasmid DNA as an input and produces a corresponding protein as an output. Only if the box functions well, we’ll have soluble proteins. If it doesn’t function well, it will fall in “N” or “I” region, and a proper stress will be applied to pull it out of the unfavorable region and push it into the favorable region. According to our previous studies, the NaCl stress effectively perturbed the metabolite composition.

## Conclusions

We partially labeled intracellular metabolites of *E*. *coli* with input ^13^C-glucose while the target protein was produced, and identified 17 metabolites. With the principal component analysis of the relative metabolite concentrations, we could designate the regions of the metabolite space according to the expression patterns of the target proteins. The regions corresponding to inclusion bodies, no expression, and soluble proteins did not separated themselves from one another, but there existed a section where the protein production was favored. We anticipate that the recombinant protein can be produced in its desired form if the metabolite profile is driven into the favorable region, by applying proper stresses. Currently, we are investigating proteins whose expression pattern and metabolite profile are changed by applying exogenous stresses.

## Supporting information

S1 File A list of genes studied in this work. (XLSX)
